# An Intuitive, Abductive Argument for a Right against Mental Interference

**DOI:** 10.1007/s10892-024-09476-7

**Published:** 2024-02-21

**Authors:** Thomas Douglas

**Affiliations:** 1 Oxford Uehiro Centre for Practical Ethics, University of Oxford, Oxford, UK; 2 Jesus College, University of Oxford, Oxford, UK

**Keywords:** Mental Integrity, Bodily Integrity, Mental Interference, Bodily Interference, Autonomy, Freedom of Thought, Mind Control, Neurorights, Manipulation

## Abstract

Several authors have recently claimed that we each possess a right against interference with our minds. However, it remains unclear how this claim is to be justified. I offer a novel argument in defence of it. The argument is intuitive—appealing centrally to intuitions regarding cases—and abductive—taking the form of an inference to the best explanation; I offer a series of cases involving intuitively wrongful interventions, argue that five somewhat promising attempts to account for the wrongfulness of these interventions leave some of this wrongfulness unexplained, and show that my proposed alternative explanation, which invokes a right against mental interference, can account for this residual wrongfulness.

It is standardly thought, in both medical and sexual ethics, that each person enjoys a moral right against interference with her body, sometimes referred to as a right to bodily integrity, or a right against bodily trespass.^1^ The existence of such a right, many would hold, helps to explain why it is generally morally impermissible to conduct medical procedures on people without their consent, to physically assault them, or to subject them to unwanted touching.

Less widely accepted is the thought that we also possess an analogous moral right against interference with our *minds*. However, several scholars have recently speculated or argued that we do possess this right (Bublitz and Merkel 2014; Douglas 2014; Douglas and Forsberg 2021; Shaw 2022; Lavazza and Giorgi 2023), or at least something in the vicinity of it, such as a (moral or legal) right against indoctrination (Murdoch 2007; Bublitz 2014; Vermeulen and van Roosmalen 2018), brainwashing (Bublitz 2014; Vermeulen and van Roosmalen 2018), unconsented-to use of neurotechnologies (Ienca and Andorno 2017), thought or mind control (McCarthy-Jones 2019), thought or mind manipulation (Bublitz 2014; Bublitz and Merkel 2014; Alegre 2017; Mendlow 2018; McCarthy-Jones 2019, Farahany 2023), intrusions in or restrictions of thought (Blitz 2010), mental influences that bypass rational control (Zohny et al. 2023), interference with our autonomous agency (Craig 2016; Roskies 2016), or interference with our thoughts (McCarthy-Jones 2019).^2^ Moreover, many more have, of course, defended broader rights that would plausibly entail something like a right against mental interference; these include rights to freedom of thought, to personal autonomy, and to respect, as well as rights of personal sovereignty and self-ownership.

Still, a moral right against mental interference is yet to attain the broad acceptance enjoyed by its bodily analogue. And, more importantly for my purposes here, our basis for endorsing this right remains unclear. Several of the authors cited above seek to derive the rights they defend from a more general right to freedom of thought or personal autonomy, but the derivation remains to be spelled out in detail. Moreover, it is not obvious, at least to this author, that such a derivation can be made to work. My own argument below will complicate any attempt to derive the RAMI from a right to autonomy, and attempts to derive it from a right to freedom of thought face the problem that the basis of this right is itself rather uncertain, at least if it is understood as a moral and not only a legal right.^3^

Nevertheless, I believe that there are sound arguments for endorsing a moral right against mental interference^4^ or ‘RAMI’, and in this article I set out what I take to be one such argument.

One way to defend a RAMI would be to shore up its theoretical grounding in, say, freedom of thought, personal autonomy or self-ownership. However, I will not pursue this strategy here. Instead, I will explore what I hope is a less theoretically fraught route to establishing the right. My argument is intuitive—relying heavily on intuitions about cases—and abductive—taking the form of an inference to the best explanation; I argue that positing a RAMI allows us to fully account for the wrongfulness of certain intuitively wrongful interventions whose wrongfulness is otherwise difficult to fully explain.^5^

As my description of this strategy no doubt suggests, I will remain neutral on what, if anything, might serve as the theoretical foundations of the RAMI. For example, I will remain neutral between the view that the RAMI is a fundamental moral right and the view that it derives from some other moral right. I will also remain neutral on what grounds moral rights more generally. I will, for instance, take no stance on whether moral rights are foundational normative features of the world, are grounded on the status (e.g., Kamm 1992) or authority (e.g., Owens 2019) that they confer on the rightholder, or are grounded on their instrumental value in protecting the interests of the rightholder or society at large (e.g., Wenar 2005).

With these qualifications in hand, we can turn to the main discussion, which proceeds as follows. I begin, in §1, by explaining why I think it is important to determine whether we possess a RAMI. Next, in §2, I offer a schematic and preliminary account of that right. In §3, I begin my argument for this right by introducing a case that will serve as the point of departure for the subsequent discussion, and by explaining why the wrongfulness of the intervention described in this case cannot be satisfactorily explained by invoking what I call a ‘right to refuse’. Then, in §§4-6, I consider three further attempts to fully account for the wrongfulness of this intervention by appealing to some further right, namely a right to control, a right to independence, a right against interference with autonomous thought or a right against interference with rational thought. I argue that, while these attempts may account for some of the wrongfulness of the intervention, they cannot account for all of it. In §7, I consider two ways of accounting for the residual wrongfulness: by invoking a right against bodily interference (‘RABI’) or a RAMI. I argue that appealing to the RABI alone cannot fully account for the residual wrongfulness, but appealing to both rights together can. Thus, positing a RAMI allows us to correct for the failings in the other attempted explanations that I have considered. Since it is not clear how else we could correct for these failings, we thus have an abductive argument for a RAMI. In §8, I introduce and respond to two objections to my argument. Then, in §9, I revisit the account of the RAMI introduced in §2. I note that my argument for the right also suggests that we would do well to revise that account in the direction of broadening its scope, and I briefly gesture towards some different ways in which we might revise it, and some challenges that we would face in doing so. In §10, I conclude.

## Motivation

I want to begin by saying something about why I think my argument is an argument worth making. More specifically, I want to say something about (i) why it matters whether we enjoy a RAMI and (ii) why we should think that any new argument for the RAMI is needed.

Concerning (i), it might seem that, once we accept—as many do—a RABI, the RAMI becomes redundant. On some views in the philosophy of mind, the mind is entirely determined by the body,^6^ or at least cannot be altered except by altering the body.^7^ The RABI provides protection against interference with those body parts that wholly determine, or are the only alterable determinants of, our minds. Thus, on these views, the RABI provides protection against interference with the mind.

It is true that, on the views in question, the RABI provides some protection to the mind. But it does not follow that the RAMI is redundant. There are two reasons for this. The first is that, over and above the protection provided to the mind by the RABI, a RAMI may provide additional protection to those body parts that determine our minds. Thus, for example, if we possess a RAMI, it may be more wrongful to interfere with those specific body parts than it is to interfere with others, since in doing so one infringes two rights, not just one.

Second, the RAMI could also provide protection against forms of treatment that are not at all protected by the RABI. This is because, even if the RABI and RAMI are attached to the same physical thing, they could protect against different kinds of treatment of that thing; what counts as a rights-infringing physical interference with that thing could be different from what counts as a rights-infringing mental interference with it. By analogy, a physical property right in a book, and an intellectual property right over its content, though in some sense attached to the same physical thing, protect against different forms of treatment of that thing: the former but not the latter protects against burning of the book; the latter but not the former protects against plagiarism of its content. Or, for a somewhat closer analogy, a right against interference with some clay that one owns and a right against interference with a statue that one owns might protect against different forms of treatment with that thing, even if the statue is made of that very clay. Slightly re-shaping the face of the statue may infringe the right over the statue, but not that over the clay; replacing bits of the statue with new pieces of clay to prevent it from deteriorating might infringe the right over the clay, but not that over the statue. Similarly, some ways of acting on the physical basis of the mind might infringe the RAMI, but not the RABI, or vice versa. For example, if we accept, as most do, that that mental states are multiply realisable by neural states, such that different neurochemical arrangements can produce the same mental states, then we must allow that an intervention could alter a person’s neural states, thus perhaps infringing the RABI, without altering her mental states, thus perhaps *not* infringing her RAMI. More importantly, in the present context, an intervention could produce only a very tiny change to a person’s neural states, thus perhaps failing to infringe the RABI, yet produce a rather major change to her mental states, thus perhaps infringing the RAMI.^8^.

The existence of a RABI does not, then, render the putative RAMI moot. The mental right could have implications that extend beyond those of the bodily right. There is thus, I think, at least a theoretical case for investing some effort in determining whether we indeed possess this mental right.

But now we need to consider (ii): why should we think that any new argument for the RAMI is needed? Isn’t it already *obvious* that we possess a right against mental interference? I do not believe that it is. Though it is true, as we have seen, that many authors have asserted the existence of something in the vicinity of this right, most of these authors have been concerned solely or primarily with a *legal* not a moral right, and the former does not obviously entail the latter. Moreover, where authors have asserted or entertained the claim that we possess something like a *moral* right against mental interference, they have not generally provided anything approaching a full defence of the existence of the right. Finally, insofar as the existing literature does suggest any defence of a moral right against mental interference, it typically suggests that this right can be derived from a more general right to autonomy. However, as my arguments below will suggest, doubts can be raised about this derivation. Indeed, in §6, I will present a case which seems to involve wrongful mental interference but seems *not* to involve any reduction of or interference with autonomy.^9^

I believe, then, that our current bases for asserting the RAMI are less sturdy than we might wish them to be. This motivates my search for an alternative case in favour of this right.

## A Schematic Account of the RAMI

Before outlining this case, I need to provide at least a schematic account of what the RAMI amounts to. There are two subquestions to consider here. What is it to possess a right against something, and what, specifically, is the RAMI a right against.

My answer to the first question is a simple one. As I use the term, you possess a right against some person treating you in some way just in case that person is under a *pro tanto* duty, owed to you, not to treat you in that way. On many accounts of rights, more is required for you to have a right. For example, on some accounts, it is necessary that the duty owed to you is absolute,^10^ is a trump,^11^ or at least is enforceable by the state.^12^ On my account, however, it is enough that the duty is owed to you.^13^

What of the second question? What does the RAMI protect against? It might be thought that the answer is easy, since it is in the name of the right: it protects against any and all interferences with a person’s mind. But that would be too quick. Consider, by analogy, the RABI. The RABI is not obviously infringed by *all* bodily interferences. There are, I think, at least two types of case in which an intervention that plausibly constitutes bodily interference plausibly fails to infringe the RABI.

First, there are cases involving very minor bodily interference. Suppose I turn on the lights, and thereby cause photons to strike your retinas. Or suppose I wave my hand near your arm, causing the hairs on your arm to quiver. In both cases, I plausibly interfere with your body, but in neither case do I clearly infringe your RAMI. The reason, I suggest, is that these interferences are trivial ones, perhaps in virtue of the magnitude of the interference falling below some threshold.^14^

Second, there may be cases in which a bodily interference fails to infringe the RABI because the relevant aspect of the right has been waived or forfeited. Suppose that, while in possession of all of my cognitive capacities and free of interference from others, I choose to attack you on the street, and you respond by physically restraining me. In this case, I take it that you interfere with my body but that you likely do not infringe my RABI, because I have likely forfeited the aspect of the right that would protect against the interference. Or suppose that, while I am in a state of delirium, you, a doctor, pin me down and inject me with a calming drug. And suppose that a short time previously, while fully competent, I had freely consented to being treated in such a way if I were to enter a state of delirium. Again, I take it that in this case, you interfere with my body, but that you likely do not infringe my RABI, because I have likely waived the aspect of the right that would protect against the interference.^15^

I will assume that similar thoughts apply to the RAMI. That is, I will allow that mental interferences may fail to infringe the RAMI when they are trivial (perhaps in virtue of falling below some threshold of magnitude)^16^ and when an aspect of the RAMI has been waived or forfeited. But I will assume that the RAMI *is* infringed by an interference with a person’s mind when (i) the interference is nontrivial, and (ii) the RAMI remains fully in force.^17^ I take (i) and (ii) to be sufficient for a mental interference to infringe the RAMI (I remain silent on whether either is necessary).

The next question to consider is ‘what counts as a mental interference?’ This is not an easy question to answer, because our intuitions—at least my intuitions—provide no clear verdict on many cases. If I cause someone to believe that it will rain shortly by electrically stimulating whatever neural circuits underpin this belief, I have, intuitively, interfered with her mind. If I cause her to form the same belief by truthfully informing her that rain is forecast by the meteorological authorities, or by drawing her attention to the dark clouds gathering upwind on the horizon, I have *influenced* her mind, but intuitively, I have not *interfered* with it. However, between these extremes, there is an expansive grey area. There is a wide range of mental influences for which the label ‘mental interference’ is neither clearly apt nor clearly inapt, and which thus cannot straightforwardly be categorised as interference (or not) on the basis of intuition alone. Examples may include altering a person’s mental states via the use of deception, threats, rhetorical techniques, framing effects, and suggestive association. The presence of this expansive grey area makes it difficult to offer a plausible and non-arbitrary criterion for mental interference on the basis of intuition alone.

How, then, are we to proceed?

One option here would be to give up on the concept of mental interference altogether and replace it with some alternative concept.^18^ The obvious candidate would be the concept of manipulation. There is plausibly a large overlap between mental interference and manipulation,^19^ and many of the ‘grey area’ examples listed in the previous paragraph are arguably forms of manipulation. Moreover, there is an extensive body of literature on the concept and wrongfulness of manipulation that we could employ to help draw a line across this grey area; many theories of manipulation give us the resources to classify these influences as wrongful or otherwise.

This will not be my strategy, however. I eschew it for three reasons. First, the boundaries of manipulation, and wrongful manipulation, are highly contested, with the result that any specific theory of manipulation that we might employ would be contentious. Second, I find it doubtful that manipulation is a sufficiently unified category for it to be plausible that we possess a single ‘right’ against it.^20^ Third, there are, I think, good *prima facie* reasons to suppose that the RAMI may be distinct from any right(s) that we do possess against manipulation. One such reason is that the bodily analogue of mental interference—bodily interference—seems to be wrongful though it is not clearly a form of manipulation. Another such reason is that the overlap between wrongful mental interference and wrongful manipulation is not obviously complete. In particular, there are, I think, some paradigmatic instances of wrongful mental interference that would not count as wrongful manipulation on at least some influential accounts of the latter.^21^ For example, if a devious neuroscientist uses some sophisticated brain stimulation technique to tinker with my beliefs without my knowledge or consent, she surely interferes with my mind, and wrongfully, but on some influential accounts of manipulation (e.g., Noggle 2020), she may not manipulate me—she manipulates me only if her tinkering causes me to have a false, irrational or otherwise criticisable mental state.

I will not, then, avoid the need to define the boundaries of mental interference by giving up on the concept altogether and replacing it with a different one. Instead, I will avoid this need in a different way. My strategy, in what follows, will be to begin by offering what I hope will be an uncontroversial sufficient condition for mental interference—a condition that captures a narrow range of interventions that *clearly* constitute mental interference. I do not think that this condition comes close to capturing the full range of mental interferences. However, it is enough to get my argument for the RAMI off the ground. Having made that argument, we will then be in a better position to consider whether a fuller account of mental interference can be defended, and I briefly return to consider that question in §9.

For the moment, I assert only the hopefully uncontroversial sufficient condition. On that condition, *A* mentally interferes with *B* when *A influences*—that is, alters^22^—at least one of *B*’s mental states and *A* does so via means that are *purely physical*, in the sense that they do not include any mental process. This condition captures at least some of what appear to be paradigm cases of mental interference—interventions such as inserting thoughts into a person’s mind using brain stimulation devices or altering a person’s affective states by administering brain-active drugs. It also chimes with the most fully developed account of the putative legal right against mental interference to have been offered to date: Bublitz and Merkel (2014: 69) suggest that *that* right also covers at least interventions on the mind that operate via purely physical means.

## The Right to Refuse

In the previous section, I suggested that the RAMI—when it has not been waived or forfeited—protects against nontrivial interferences with a person’s mental states, and that one interferes with another person’s mental states (at least) when one influences her mental states via purely physical means.

Having thus schematically characterized the RAMI, I am now in a position to begin my defence of it. This defence will be organised around a central case. I will argue that several initially promising attempts to account for the wrongfulness of the actions described in this case, and a number of variant cases, fail to fully account for this wrongfulness. I will then propose that the residual, unexplained wrongfulness can be accounted for if we endorse a RAMI.

Here is the case that will serve as my focal point:

*Brain Stimulation.* Your housemate, Lucien, has been feeling a little gloomy; much of his mental life has acquired a slightly more negative quality than it had previously. Activities that he previously enjoyed give him a little less pleasure, pain feels a little bit worse, and so on.^23^ You think he might benefit from speaking with a counsellor, and you’ve tried convincing him to do so, but he has proved resistant. So you take it upon yourself to put an end to his gloominess. While he is sleeping, and without his knowledge or agreement, you place electrodes on his head and administer a small electric current to the mood centres of his brain for a few minutes. This significantly lifts his mood for a week. Your intervention has no other mental or physical effects—that is, no effects not mediated by the diminished gloominess. Moreover, you knew in advance that it would have no such effects.^24^

It seems to me that you wrong Lucien in *Brain Stimulation*.^25^ But how do you wrong him?

One suggestion might be that you wrong him by infringing an autonomy-right of Lucien’s: his right to be given the opportunity to block your intervention by withholding his consent to it—henceforth, his ‘right to refuse’. You wrong him by failing to give him the opportunity to block your intervention in this way—henceforth just ‘the opportunity to refuse’.

This suggestion is, it seems to me, correct. But it is only part of the story. There are many ways in which we can affect one another’s lives, and indeed influence their minds, without giving them the opportunity to refuse, but also without wronging them. Suppose that, unannounced, I approach a stranger on the street and offer a compliment on his outfit, or admonish him for dropping litter, or recommend that he look at the beautiful sunset. In each case I alter his mental states. And in each case, I do so without giving him the opportunity to block my action by withholding consent. Yet in none of these cases do I wrong him.

Thus, it seems that our right to refuse has its limits. It covers some forms of treatment, but not others. What explains this? A plausible answer is that the right to refuse is a parasitic right. We possess various rights against being treated in certain ways. At least some of those rights are rights that we can waive by consenting to a form of treatment that would otherwise infringe the right. However, in the absence of our issuing a such a waiver, those who treat us in the ways prohibited by the right infringe it. Thus, we enjoy a derivative right to block this form of treatment by refusing the waiver.^26^

The question then becomes: on what right is Lucien’s right to refuse your brain stimulation intervention parasitic? Which deeper right of his do you infringe by nonconsensually stimulating his brain as you do? In the subsequent sections, I will consider three answers to this question. Each answer appeals to a right or rights that, like the right to refuse, might aptly be described rights *to autonomy*. But the three answers differ in what they take autonomy, or the right to it, to consist in.

## The Right to Control

The first answer holds that we each have a right that others not reduce our bodily or mental autonomy—our autonomy with respect to (certain aspects of) our bodies or minds, such as our thoughts, feelings, or actions. It further holds that a person enjoys autonomy with respect to an aspect of her body or mind (henceforth just ‘self’) to the extent that she has control over it.^27^ Thus, we each have a right that others not reduce our control over (certain aspects of) our selves. You wrong Lucien, according to this answer, by doing precisely that.

The problem with this answer is that it is not clear that you do reduce Lucien’s control over any aspect of his self. We can note, to begin with, that you do not clearly diminish Lucien’s *global* control over his self. Perhaps his gloominess was functioning as a constraint on his control over many aspects of his self, preventing him from, say, performing certain actions, thinking certain thoughts, or feeling certain feelings. And perhaps, by easing this constraint, you have enhanced his overall control over his self.

Of course, there could still be *aspects* of his self over which Lucien has lost some of his control. Perhaps, for example, he has lost some control over how gloomy he feels. However, if we take a long-term perspective, even that is not clear. It may be that, by initially diminishing his gloominess, you will cause Lucien to enter a reflective and clear-thinking phase of his life in which he finds he is able to modify his mood at will. In that case, the overall effect of your intervention on Lucien’s control over his gloominess may be not to reduce it, but to enhance it.^28^

What is more plausible is that you diminish Lucien’s control over his gloominess *locally*—by which I mean his gloominess during some restricted time period. Perhaps—to take this approach to its extreme—we could say that you reduce Lucien’s control over how gloomy he feels at precisely that instant at which your interference first exerts its gloominess-mitigating effect. It may be that, at least at that very moment, Lucien is less able to maintain a high level of gloominess than would otherwise have been the case. And perhaps this is enough to make your intervention wrongful.

However, it is possible to imagine versions of *Brain Stimulation* in which Lucien does not suffer even this reduction of control, yet your brain stimulation procedure remains intuitively wrongful. We simply need to stipulate that, even in the absence of your intervention, Lucien’s gloominess would have been beyond his control. Perhaps, for example, the gloominess was triggered by a brain tumour caused by random genetic mutations. And perhaps this tumour causes him to experience some fixed level of gloom which he is powerless to alter and does not endorse. Your brain stimulation procedure then reduces Lucien’s gloom to a lower level. Call this variant of the case *Tumour*. Since, in *Tumour*, Lucien in any case fully lacks control over how gloomy he feels, you have done nothing to diminish his control, even locally. Nevertheless, it remains plausible that you have wronged Lucien. We cannot account for this by claiming that you have reduced Lucien’s control over (some aspect of) his self, since you have not—he already lacked the relevant control.^29^

## The Right to Independence

How can we explain that element of the wrongfulness of your intervention in *Brain Stimulation* that persists in *Tumour*? One suggestion would be that we can do so by moving to a somewhat different understanding of autonomy, and/or of the right to it. For the moment, let us hold to our conception of the right to autonomy—we have a right that others not reduce our autonomy with respect to (certain aspects of) ourselves—but reconsider our conception of autonomy itself.

In the previous section, I equated autonomy with control. But autonomy is sometimes taken to require not, or not only, control, but also the absence of certain kinds of influence by others—what Raz (1986: 377–378) calls *independence*. On views of this kind, one way to reduce a person’s autonomy—and hence to infringe their right to autonomy, as we are currently understanding it—will be to reduce the independence of (certain aspects of) their selves. How might one do that? Different views are available, but on the one that I think is most conducive to explaining the wrong in *Tumour*, you lack independence with respect to some aspect of yourself insofar as it is the product of *interference* by another.^30^ Thus, one might infringe another’s right to autonomy by interfering with some aspect of their self, thereby increasing the degree to which it is the product of interference by others.

Consider how this view may play out in the case of *Tumour.* As we have seen, you do not, in *Tumour*, diminish Lucien’s (global or local) control over how gloomy he feels, because his gloominess would not, in any case, have been within his control. Nevertheless, it is plausible that you do yourself *interfere with* his gloominess, and that his resultant lower level of gloominess is, at least in part, the product of that interference. It may be, then, that you diminish his independence with respect to his gloominess. Thus, if Lucien enjoys a right that others not reduce his independence with respect to his gloominess, you will have wronged him.^31^

Difficulties again arise, however. We can imagine variants of *Brain Stimulation* in which at least part of the wrongfulness present in the initial case persists, yet we cannot explain this by adverting to a reduction in independence. Suppose that Lucien’s gloominess, prior to your intervention, is itself wholly the product of interference by others. Suppose that a devious barista has been spiking Lucien’s post-lunch coffees with a gloom-inducing drug—a drug that produces, without any mediating thought on Lucien’s part, a feeling of gloom which Lucien does not endorse. Your brain stimulation procedure then diminishes Lucien’s level of gloominess from this barista-induced baseline to a lower level.

In this variant of the case, which I will call *Spiked Coffee*, your action does not in any way increase the degree to which Lucien’s gloominess is the product of interference by others. It will in any case be wholly the product of such interference. Yet your action still seems wrongful. We cannot account for its wrongfulness by adverting to the loss of independence that it causes; it causes no such loss.

## The Rights Against Interference with Autonomous Thought and Against Interference with Rational Thought

It might be thought, however, that we can accommodate the wrongfulness of your action in *Spiked Coffee* by shifting our focus from the way in which interference reduces one’s independence—and thus *ceteris paribus* one’s autonomy—to the interference itself. We might claim that you wrong Lucien not by increasing the degree to which some aspect of his self is the product of interference by others, but simply by interfering with that aspect. Perhaps Lucien has a right against interference with certain aspects of his self. You infringe that right by interfering with those aspects. And you do so even if those aspects would in any case have been the product of interference by others, so not independent.

This suggestion is, I think, correct. But it invites a further question. *Which* aspects of Lucien’s self are covered by this right against interference?

One plausible answer would again invoke the concept of control. One might claim that Lucien enjoys a right against interference with exercises of his capacity for control—with what I will call his ‘autonomous thought’. Another answer would appeal to rationality. One might claim, for example, that Lucien enjoys a right against interference with exercises of his capacity to respond to reasons—with what I will call his ‘rational thought’.^32^ Either way, it will be plausible that your intervention infringes Lucien’s right. Regardless of your intervention, Lucien’s level of gloominess in *Spiked Coffee* will not itself be the *product* of his own autonomous or rational thought (henceforth ‘A/R thought’); it will instead be the product of interference by either you or the barista. However, even following the barista’s intervention, and yours, Lucien plausibly still possesses the capacity for control and for reasons-responsiveness. Moreover, his level of gloominess may affect how he will, in the future, exercise those capacities. Thus, by reducing his gloominess, you may affect his future A/R thought. For example, you may alter his ends—the values, commitments and desires that he will seek to satisfy or fulfil through his A/R thought. Perhaps, by diminishing Lucien’s gloominess, you will contribute to his forming desires to reconnect with his friends, take up a hobby, and reset his career. And perhaps he will now invest much of his A/R thought in the pursuit of those ends. If so, you will arguably have interfered with his A/R thought. Of course, his A/R thought would in any case have been interfered with by the barista, but on the present view, that does not prevent you from wronging him. You wrong him simply by interfering with his A/R thought, not by increasing the degree to which it is the product of interference.

However, while it is possible that you interfere with Lucien’s A/R thought in *Spiked Coffee*, we can, again, concoct variants of this case in which you do not do so. Consider yet another variant of *Brain Stimulation*, which I will call *Dream Modulation*. In this variant of the case, as in *Spiked Coffee*, a barista has been spiking Lucien’s drinks, causing him to experience gloominess, and you subject him to an intervention which mitigates that gloominess. But in *Dream Modulation*, the barista-produced gloom afflicts Lucien only during dreams of a certain type—dreams that the barista’s drug itself causes him to experience. These dreams lack any narrative arc or linguistic content. In them, Lucien experiences himself to be lying on his back looking upwards at clouds blowing across the sky above him, while feeling somewhat gloomy. Your brain stimulation procedure does not prevent these dreams but does cause their affective quality to be a little less negative. Suppose, moreover, that, in part because Lucien never remembers these dreams, your intervention has no effect at all on his wakeful mental life.

In this case, we cannot with any plausibility say that you have altered Lucien’s ends—the values, commitments and desires that Lucien will autonomously pursue. Nor can we plausibly say that you have interfered with his A/R thought in any other way, for you have affected his mental life only during periods in which he is engaged in no A/R thought.

Nevertheless, in subjecting Lucien to your dream-modulating brain stimulation procedure without his consent, you surely still wrong him, at least mildly; it was not your place to nonconsensually intervene on his dream life in this way. And we cannot account for this wrongfulness by claiming that you have infringed either his right against interference with autonomous thought or his right against interference with rational thought.

## Accounting for the Residual Wrongfulness

Still, you clearly do interfere with some aspects of Lucien’s self in *Dream Modulation*. For example, you interfere with his gloominess, and with the neural correlates thereof. Thus, we might suspect that it should be possible to account for the wrongfulness of your intervention by invoking some kind of right against interference. But a right against interference with *what*, exactly?

There are, I think, two obvious candidates: a right against interference with the body, and a right against interference with the mind; we might seek to explain how you wrong Lucien in *Dream Modulation* by claiming that you infringe either his RABI or his RAMI.

Consider first the possibility that you wrong Lucien by infringing his RABI. It seems to me that you *do* wrong him in this way. The mere placing of electrodes on Lucien’s scalp without his consent is, I think, enough for you to infringe his RABI. That you also alter his bodily (neural) states through applying an electric current to his brain makes it yet clearer that you infringe this right.

It is, however, doubtful that your infringement of Lucien’s RABI fully accounts for the wrongfulness of your intervention.

One reason for this is that the mental impact of your intervention seems more significant than the bodily impact, so it would be puzzling if the bodily impact did all of the work in explaining the wrongfulness of the intervention. Another reason is that ‘subtracting’ the mental effects of *Dream Modulation*, while holding the magnitude of the bodily effects fixed, seems to alter our moral appraisal of the intervention. Consider a variant of the *Dream Modulation* case in which your brain stimulation procedure causes neural changes in Lucien that are similar in magnitude to those that you caused in the original version of the case, but suppose that these neural changes have no mental effects: they affect neither his gloominess nor any other aspect of his mental life. Call this case *Mentally Inert Neuromodulation.* (*Mentally Inert Neuromodulation* may be a scientifically fantastic case, but it is not metaphysically fantastic; as noted earlier, it is standard to accept that mental states are multiply realisable by neural states, such that different neurochemical arrangements can produce the same mental states. So, in principle, one can modify a person’s neural states without producing any mental change.)

It seems to me that the wrong that you perpetrate against Lucien in *Mentally Inert Neuromodulation* is less serious than, or at least qualitatively different to, the wrong that you commit in the original *Dream Modulation* case. In particular, your intervention in *Mentally Inert Neuromodulation* seems in one important way less intrusive than that in *Dream Modulation.* Yet the bodily impact is similar. This suggests that we cannot fully account for the wrongfulness of your action in *Dream Modulation* by adverting to the bodily impact of your action.

How could we account for the residual wrongfulness of your action in *Dream Modulation*? That is, how could we account for the wrongfulness of your action in that case that is not shared with *Mentally Inert Neuromodulation*? One way to do so would be to invoke a right against *mental* interference—a right against interference with one’s mind. If Lucien possesses such a right, you clearly infringe it in *Dream Modulation*. On the other hand, you clearly do not do so in *Mentally Inert Neuromodulation.*

Recall that, in §2, we specified that *A* interferes with *B*’s mind if *A* alters *B*’s mental states and *A* does so via purely physical means. Further, we specified that, if this interference is nontrivial and no aspect of the RAMI has been waived or forfeited, then it infringes *B*’s RAMI, supposing that *B* possesses such a right. In *Dream Modulation*, you alter Lucien’s mental states, and you do so via purely physical means: the administration of an electric current directly to the brain. Moreover, the interference is plausibly nontrivial, and there is no reason to suppose that Lucien has waived or forfeited any aspect of his RAMI. On the other hand, in *Mentally Inert Neuromodulation* you do not interfere with Lucien’s mind at all, since you do not alter Lucien’s mental states.

The right against mental interference thus allows us to account for the wrongfulness present in *Dream Modulation* that is not shared with *Mentally Inert Neuromodulation.* Moreover, it is rather unclear how else we could account for this residual wrongfulness (though I will consider some alternative possibilities in the next section). After all, as I have already noted, you do not, in *Dream Modulation*, reduce Lucien’s control or independence with respect to any aspect of his self, and you do not interfere with his autonomous or rational thought. If we indeed lack an alternative explanation for the residual wrongfulness in *Dream Modulation*, we have an abductive argument for the right against mental interference.

## Objections

How might an opponent of the RAMI respond? I suspect that they would do so in one of two ways: by maintaining that accepting a RAMI would have unacceptably counter-intuitive implications, or by furnishing an alternative explanation for the residual wrongfulness present in *Dream Modulation* (that is, the wrongfulness not shared with *Mentally Inert Neuromodulation*). In this section, I consider these two objections in turn.

Consider first the claim that accepting a RAMI would have unacceptably counter-intuitive implications. This objection will, I think, have greatest force when grounded in an ‘extended’ account of the boundaries of the mind. On such accounts, certain cognitive aids that are external to our bodies—such as our books, diaries and smartphones—have just as strong a claim to being among the physical bases of our minds as do our brains.^33^ Put somewhat less formally, extended accounts of the mind hold that our minds reside just as much in these external cognitive aids as they do in our brains. As many have noted, such accounts have interesting, and in many cases challenging, potential ethical implications,^34^ and it might be thought that they pose a particular challenge to proponents of the RAMI: if an extended account of the mind holds, then it looks as though re-arranging the frequently consulted books in someone’s bookshelves or tearing a page out of her diary would count as an infringement of the RAMI in just the same way as would biochemically altering her neural states. This, it might be thought, is implausible.

My preferred response to this objection is simply to bite the bullet. It seems to me easier to accept that one *is* wronged by having one’s diary or smartphone or other external cognitive aids interfered with than to give up on the RAMI, and thus to give up on fully accounting for our intuitions concerning *Brain Stimulation* and its variants. However, to those whose intuitions incline them in the other direction, I offer an alternative, more concessive response: if an extended account of the mind holds, then we can concede that there is no RAMI, where that is understood as a right against interference with *any part of* our minds, but maintain that we do enjoy a right against interference with *the non-extended parts of* our minds. That is, we can simply restrict the scope of the right so that it does not cover (all) aspects of the mind that are ‘housed’ in external objects. After all, the external mind thesis is a view about what the natural boundaries of the mind are, not about where the boundaries of the mind’s moral protection should be set. We are free to set the boundaries of that moral protection more narrowly than the boundaries of the mind itself.

Consider now the second objection, according to which there are alternative ways of accounting for the residual wrongfulness of your action in *Dream Modulation*. I can see two different ways in which this objection might be developed. First, it might be held that Lucien possesses a right not against interference with *any* part of his mind, but only against interference with some specified aspects of his mind.

The difficulty here is that the parts of the mind that most plausibly deserve the protection of a right against interference do not plausibly include the dream states with which you interfere. We have already considered the possibility that Lucien possesses a right against interference with his autonomous thought or against interference with his rational thought. However, as we noted, such a right could not account for the wrongfulness of your action in *Dream Modulation*, in which Lucien’s autonomous and rational thought are both left unscathed. I believe that the same problem will arise in relation to all other aspects of the mind that might plausibly enjoy special moral protection. For instance, though it is, I think, somewhat plausible that Lucien enjoys a special right over those parts of his mind that are authentically his, that constitute his true self or that are central to his narrative identity, it is not plausible that his drug-induced and never-recalled dreams fall within these parts of his mind.

The second, and I think more promising, way of developing this objection would be to hold that, though Lucien’s whole mind enjoys the protection of some right, that right is not a right against *interference*—at least, not as I have characterized interference (viz. as the alteration of mental states via purely physical means). Rather, the objector might maintain, the relevant right over the mind is a right against some other type of influence. For example, perhaps Lucien possesses a right against influences that constitute *control* of his mind by the influencer,^35^ that are hard for him to avoid,^36^ or that fail to engage his autonomous or rational (A/R) thought.^37^ All of these putative rights would also plausibly be infringed by your intervention in *Dream Modulation*, so it might be thought any one of these rights could be invoked to explain the residual wrongfulness of your action in that case.

My response is to claim that each of these rights either fails to explain the residual wrongfulness of your action in *Dream Modulation*, or itself entails a right against mental interference.

Consider first the suggestion that you wrong Lucien by infringing a right against influences that *control* his mind. I think it is doubtful that this right can explain the wrong present in this case since it seems that the wrong would persist even if you do not, in stimulating Lucien’s brain, control any aspect of Lucien’s mind. To control something is, among other things, to have the ability to relatively finely and reliably determine the state of that thing.^38^ But suppose that you lack this ability with respect to Lucien’s gloominess. The brain stimulation procedure is rather hit and miss. It sometimes reduces a person’s gloominess, but often fails, and when it does diminish gloominess, it sometimes does so to a high degree, and sometimes only to a low degree. It seems to me that, were you to employ this fickle brain stimulation procedure on Lucien, and to succeed in mitigating his gloom, the wrongfulness of your treatment of him would be undiminished, even though you could not aptly be described as controlling his gloominess.

Similar thoughts apply to the suggestion that you wrong Lucien by infringing a right against hard-to-avoid influences on his mind. Suppose that Lucien knows from the start about your plan to stimulate his brain while he sleeps, and could easily have avoided the influence simply by locking his bedroom door, but he never got around to doing this. In this variant of the case, your influence is not hard to avoid. But it still seems just as wrongful.

What of the suggestion that you wrong Lucien by infringing a right against influences that do not operate by engaging the influencee’s A/R thought—henceforth, *bypassing* influences? This suggestion is, I think, quite promising: I think it is plausible that Lucien does possess such a right, and that your brain stimulation procedure infringes it. However, note that a right against bypassing mental influences will itself entail a right against mental interference, as I have been understanding mental interference here. Mental interferences operate via purely physical means. But this means that, if a mental influence is to count as a mental interference, it cannot operate by engaging A/R thought, since autonomous and rational thought are mental, not purely physical, processes. Thus, mental interferences are a subset of the category of *bypassing* influences. So while I am open to the possibility that we can explain the wrong in *Brain Stimulation* by appealing to a right against bypassing influences, I do not see this explanation as in tension with an explanation adverting to a right against mental interference. Rather, it simply subsumes that explanation by positing a broader right.

Indeed, in the next section I will suggest that there may be a case for collapsing the distinction between these rights by broadening our understanding of mental interference so as to include all—or at least a wider range of—bypassing mental influences, not merely those that operate via purely physical processes.

## A Broader Conception of Mental Interference?

Let me introduce one last case. Suppose that all is as in the original version of *Dream Modulation*, except that now you diminish the gloominess of Lucien’s drug-induced dreams not through an electrical brain stimulation procedure, but in the following way: while he is sleeping you play a sound that resembles the sound of a buzzing lamp that you know was present when Lucien received some good news recently. You suspect, correctly, that Lucien will subconsciously associate the sound with euphoric feeling that he experienced when he received the news, causing him to experience an attenuated version of the euphoria during his dream. Call this version of the case *Buzzing Sound*.

*Buzzing Sound* does not involve mental interference, as I have been characterizing it, since the subconscious associative process via which it operates is—I take it—a mental process. Nevertheless, it strikes me as intuitively plausible that *Buzzing Sound* does involve mental interference. Moreover, it might seem that your intervention in *Buzzing Sound* shares at least some of the wrongfulness present in the original version of *Dream Modulation*. This might lead us to suppose that we should revise our understanding of mental interference, and of the right against it, so as to cover cases like *Buzzing Sound.* This, however, quickly leads us into thorny territory. By virtue of what, exactly, does your intervention in *Buzzing Sound* count as a mental interference? The answer is not clear.

One possibility, already alluded to at the end of the previous section, is that it constitutes a mental interference by virtue of being a bypassing influence—an influence that does not operate by engaging the A/R thought of the influencee. But this quickly raises a number of difficult boundary-setting questions. These include questions about which thought processes, exactly, are rational ones, a matter on which there is considerable disagreement.^39^ They also include questions about what it takes to engage those processes. Some suggest that it requires that the influencer influences by giving or communicating reasons (Shiffrin 2000: 213; Seymour Fahmy 2011: 178; Levy 2019). But this suggestion is open to multiple interpretations. Must giving or communicating a reason involve explicitly stating a proposition concerning the influencee’s reasons, or may the proposition be implicitly expressed, for example, though asking a pointed question or frowning in response to a suggestion? And how, exactly, must the influencee respond? For example, must the reason-receiver *explicitly* recognise the reason or is it enough that she responds to it in a way that somehow *tracks* the reason? And then, once we have settled the question of what it takes to engage rational thought, we must turn to consider the analogous, and equally challenging, question concerning *autonomous* thought.

Further difficult questions concern the plausibility of the suggestion that all bypassing mental influences count as mental interferences and so as potential infringements of the RAMI. This, it might be thought, would leave us with an over-broad right.^40^ There are many rather mundane forms of mental influence that seemingly bypass both autonomous and rational thought, yet seem rather morally innocuous, even when they are nontrivial and where it is plausible that the RAMI remains fully in force; consider, for example, the act of wearing perfume and thereby eliciting feelings of attraction in one’s date.

It was for reasons such as these that I initially shied away from a broad characterisation of mental interference, and thus of the right against it. However, we will ultimately need to confront these matters. The argument for the RAMI that I have given in this paper can seemingly be generalised to some cases of mental influence that do not operate via purely physical means, and any fully adequate account of the RAMI will need to do justice to that. It will ideally, I suspect, need to specify the scope of the RAMI such that it includes cases like *Buzzing Sound*, yet avoids including interventions that are, intuitively, morally innocuous. I leave this as a challenge to be confronted in future analyses.

## Conclusion

I have offered a novel argument for the view that we possess a RAMI. More specifically, I have tried to show that we need to posit such a right to fully explain the wrongfulness present in *Brain Drug* and its variants.

I hope that, in providing this argument, I have helped to provide the RAMI with a more secure justification than it so far enjoys. Existing justifications for this right standardly claim that it can be derived from the value of, or a right to, autonomy. But it is not clear how this derivation is supposed to work, and moreover, my own argument casts doubt on some ways in which it might be thought to work: I have argued that the wrongfulness of some mental interferences cannot be grounded in either the claim that they reduce autonomy or interfere with autonomous thought, for these interferences do neither.^41^

Of course, my own justification is not a fully satisfying replacement for autonomy-based defences, since it—being an abductive argument—it does not itself provide the RAMI with theoretical foundations. We may still wonder: *by virtue of what* do we enjoy the RAMI? This, again, I must leave as a challenge to be confronted in future analyses.

## Footnotes

1 For examples of the ‘trespass’ terminology, see Thomson (1990) and Archard (2008).

2 For a critical discussion of several of these proposals, see Zohny et al. (2023).

3 For discussion of some of the difficulties, see Schauer (2020).

4 I henceforth generally omit the qualifier ‘moral’. I insert the qualifier ‘legal’ whenever I wish to refer to the legal right against mental interference.

5 My strategy here parallels one offered by others in support of some similar rights or principles. See, for example, Ripstein’s (2006) argument for the sovereignty principle, and Archard’s (2008) argument for the wrong of bodily trespass. For critical discussion of this strategy as it has been employed in defence of self-ownership, see Aas (2023).

6 Here, I am thinking of so-called internalist views of mind, on which “an individual’s mental states are fixed or determined by the intrinsic, physical properties of that individual” (Wilson 2017). Versions of internalism are defended in Stich (1978), Fodor (1980), and Segal (1991).

7 Here I am thinking of views on which an individual’s mental states are determined in part by features of the world beyond that individual, but on which those external features are social conventions about meaning, facts about our evolutionary history, or other features of the world that individuals are not normally able to alter. For the view that mental states are determined in part by social conventions, see, for example, Burge (1979). For the view that mental states are determined in part by our evolutionary history, see, for example, Papineau (2022).

8 I will revisit this issue in §7.

9 The case does involve an infringement of what might aptly characterised as an autonomy-right—the right to determine, through giving or refusing consent, whether the interference occurs. But this right is, I will suggest, parasitic on something like the RAMI.

10 Nozick (1974) is often understood as holding that the duties corresponding to rights are absolute. See, for example, Francis and Francis (1976). Others attribute to Nozick the slightly weaker view that the duties corresponding to rights are almost absolute, on the basis that, in a footnote, Nozick leaves open the possibility that rights might be permissibly infringed in order to avoid “catastrophic moral horror” (1974: 30). See, for example, Vallentyne (2011) and Arneson (2011).

11 See, for example, Dworkin (2013: 6). I take the claim that a duty is a ‘trump’ to mean that it can only be defeated by other duties that are also trumps.

12 See, for example, James (2003) and Flanigan (2019).

13 Because the duty that corresponds to a right is, on my view, a *pro tanto* (that is, defeasible) duty, it can sometimes be morally permissible, all things considered, to infringe a right. Nevertheless, on my terminology, infringing a person’s rights does necessarily *wrong* that person, and is *wrongful*. To wrong someone, or treat them wrongfully, is, on my usage, simply to fail to live up to a *pro tanto* duty owed to that person—that is, to infringe some right of hers. I am here borrowing my terminology from, among others, Kamm (2004: e.g., 477-8).

14 I will not take a firm view on what determines the magnitude of a bodily interference, nor on where the threshold for (non)triviality lies, but I take it to be plausible that the magnitude of a bodily interference will be determined by, perhaps among other things, the proportion of the body that is interfered with, the importance of the interfered-with body parts, and the degree of alteration to or destruction of those body parts.

15 It might be suggested that there is no infringement of the RABI in these cases because there is no bodily interference. Bodily interference entails, it might be thought, that the right against it remains in force. I acknowledge that there may be a concept of bodily interference on which this is so. But I think that there is also a less normatively loaded concept of bodily interference which has no such entailment—a concept on which we can, for example, perfectly well speak of a validly consented-to medical intervention as a form of bodily interference, though it infringes no bodily rights. I am interested in the latter concept of bodily interference, and the analogous concept of mental interference.

16 I leave it open what would determine the magnitude of a mental interference, but we might suppose that this will depend on how many mental states or parts are interfered with, on the centrality and/or importance of those mental states or parts, and on the magnitude of any interferences with them.

17 When I state that a right remains fully in force, I mean simply that no aspect of it has been waived or forfeited.

18 I thank an anonymous reviewer for pressing me to consider this option.

19 It is for this reason that I above listed claims to the effect that we possess a right against manipulation when listing assertions of rights *in the vicinity of* the RAMI. On what is arguably the dominant account of manipulation, what I will call mental interference is plausibly a subtype of manipulation. I am thinking here of the view that manipulation consists in the bypassing or subversion of rationality. (For the claim that this is a dominant view, and for critical discussion of it, see, for example, Gorin (2014) and Gibert (2023).) I suspect that everything that counts as mental interference, on my view, also counts as bypassing rationality, though it is difficult to be sure, since precise conditions for bypassing rationality are seldom given.

20 I am inclined to agree with Blumenthal-Barby (2014: 122) when she writes that “it is not the case that any of [the] practices traditionally labeled as ‘manipulation’ are ipso facto morally wrong; nor is it even the case that any of these practices always has a single wrong-making feature”, and with Gibert (2023: 336) when she claims that “[t]here is no need to identify a common feature shared by all instances … of wrongful manipulation, for there is no unified, non-morally specifiable feature to be found”.

21 I will not commit myself to a particular account of manipulation, or to any view regarding the relationship between mental interference and manipulation. I will, however, sometimes draw on extant attempts to explain the wrongfulness of manipulation in seeking to explain the wrongfulness of my central cases of mental interference.

22 I leave open various questions that would need to be answered regarding what it takes to *alter* a person’s mental states. For example, must one *cause* the alteration, or is it enough that one allows it to occur? And must the alteration be intended, foreseen, foreseeable, or none of the above? I will focus on cases that count as ‘altering’ on any plausible understanding thereof.

23 As my comments here indicate, I will be thinking of Lucien ‘gloominess’ as a purely affective phenomenon that need not have any cognitive or conative elements or effects.

24 In this case, and all variants of it given below, I assume that you—the protagonist—have perfect knowledge of the intervention’s effects.

25 In holding that you wrong Lucien, I do not mean to imply that you perform an action that *is wrong*—where that would imply that it is, *all things considered*, impermissible.

26 For a similar point, see Ripstein (2006: 221).

27 For examples of authors who assert control to be central to autonomy, see Arpaly (2003: 118); Raz (1986: 269); Taylor (2009: 6–7).

28 Blumenthal-Barby (2021: 71) makes a similar point.

29 As Pugh (2020: 81–82) points out, *not reducing* a person’s autonomy is consistent with wronging a person by failing to increase her autonomy. However, in *Tumour* it seems that you wrong Lucien even if there is nothing else you could have done that would have left him with more control over his gloominess. Thus, we cannot fully account for the wrongfulness of your action in *Tumour* by appealing to your failure to increase Lucien’s control.

30 An influential alternative version of the view specifies that you lack independence with respect to some aspect of yourself to the extent that it is the product of coercion or manipulation. See for example, Raz (1986: esp. 377-8). Other alternative versions (e.g., Pettit 1997; Sparrow 2014) allow that one’s independence can be reduced by the mere fact that another has the *ability* to influence an aspect of your life in a certain kind of way; your independence is threatened even if that ability is not exercised.

31 For critical discussion of a similar objection to nudging, see Blumenthal-Barby (2021: 95–96).

32 This answer is suggested by the literature on paternalism, much of which takes interference with rational agency to be central to wrongful paternalism. Cholbi (2017), for instance, characterizes paternalism as involving “unauthorized intercessions in the operations of their targets’ rational will” (124). See also Shiffrin (2000).

33 For the classic defence of this view, see Clark and Chalmers (1998).

34 For discussion of some of these, see Anderson (2008); Buller (2013), Bublitz and Merkel (2014), Glannon (2014), Levy (2007), and Lippert-Rasmussen (2018).

35 For the suggestion that influences can be wrongful by virtue of constituting interpersonal control, see, for example, Faden et al. (1986: esp. 238–241, 256–260), and Zuboff (2020).

36 For discussion of this possibility in relation to nudging, see Kipper (2021) and Saghai (2013).

37 For discussion, see, for example, Gorin (2014) and Levy (2019).

38 For discussion of this point, see De Marco and Douglas (in press).

39 For a summary of some of the disagreement, especially insofar as it bears on the ethics of nudging, see Schmidt (2019: §§III-IV).

40 For some putative counterexamples to it, see Gibert (2023: §II.B).

41 This is consistent with the RAMI being grounded in autonomy in some more complicated way. For example, nothing I have said precludes the possibility that we possess the RAMI in virtue of our status as autonomous agents, or in virtue of the normative autonomy that the RAMI confers on us, or in virtue of the fact that the mental interferences *very often* diminish our autonomy.
